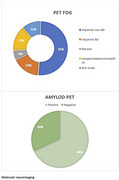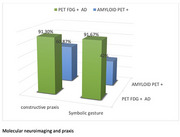# Praxis in dementia: Could be predictors of biology?

**DOI:** 10.1002/alz70861_107931

**Published:** 2025-12-23

**Authors:** Daniela Castro, Shenda Orrego, Mihaela Sava, Maria Delso, Maria Pilar Garcia, Amaia Mari, Carmen Terron, María Sagrario Manzano

**Affiliations:** ^1^ Psychiatric Department, Infanta Leonor Hospital, Madrid, Madrid Spain; ^2^ Universitary Hospital Infanta Leonor, Madrid, Madrid Spain; ^3^ Psychiatric Department, Madrid Spain; ^4^ Psychiatric Department, Hospital Infanta Leonor, Madrid Spain; ^5^ Psychiatric Department, Infanta Leonor Hospital, Madrid Spain; ^6^ Head of Nuclear Medicine Department, Universitary Hospital Getafe, Madrid Spain; ^7^ Nuclear Medicine Department, Universitary Hospital Gregorio Marañon, Madrid Spain; ^8^ Neurology Department, Nuestra Señora del Rosario Hospital, Madrid Spain; ^9^ Neurology Department Infanta Leonor Hospital, Madrid, Madrid Spain

## Abstract

**Background:**

**molecular imaging is** essential in order to make a diagnosis with a high level of accuracy in patients with cognitive and/or behavior complaints. Neuropsychological assessment, such as praxis, in patients with cognitive impairment could be predictors of these results.

**Method:**

retrospective and descriptive study in patients with Amyloid PET (APscans) implemented according to rational use of this technic, between January 2019‐october 24 in Neurology Department, Infanta Leonor Hospital, Madrid, Spain. We included: first visit date, type of diagnosis, date, age, sex, neuropsychiatric symptoms‐NPSq‐(psychosis, depression, behavioral alterations, sleep disorders, anxiety, suicidal ideation), doctor requesting the diagnosis, CT/MRI scans (atrophy or vascular signs), NPS values (FOTOTEST, MMSE, MOCA, praxis), GDS, amyloid‐PET scans, 18F FDG PET/TC scans (AD pattern; non‐AD pattern; normal, indeterminate). The study was approved by the Research Committee of ILH. Statistical analysis was made with Dataset and SPSS 22.0

**Result:**

total of 72 patients (54,17 %females) with mean of age:61.75+/‐7.79 years old. Vascular risk factors:56.94 %. GDS 3=75.71%. 68.06% of patients come from General Practitioners. NPS results: MOCA=25.41; MMSE=26.07; FOTOTEST=31,49. 49.30 % patients have history of depression, and suicidal ideation:50%. Symbolic gesture impaired:31,29%; constructive praxis impaired:34.85%. 48.61% of patients have a diagnosis of MCI pre‐PET studies. Post‐PET the diagnosis of EA was 36,12%. In structural neuroimaging we observed 33.80% of patients with atrophy in MRI scans. A vascular pathology in MRI:27,94%. 18F FDG PET/TC scans were not normal in 68.8%. Within these cases, 51,39% 18F FDG PET/TC have a not AD pattern, being indeterminate in 15.28%. Positive AP Scans: 36.11%. In 23.08% the results were not conclusive and the patients remains without a diagnosis in the follow up. Positive APScans has depression in 34,29% and psychosis in 66.67%. If you take into account only praxis, the probability of APScan positive is four times. In a multivariant model, constructive praxis together with neuropsychiatric symptoms in general, and depression, ovel all, increase 20 times the probability of a positive APScan.

**Conclusion:**

In patients with cognitive and behavior complaints, biomarkers are essential to make a diagnosis. The combination of constructive praxis together with NPSq, and, over all, depression, increase 20 times the probability of a positive APScan.